# ﻿A new species of *Galathea* (Decapoda, Galatheidae) from the seamounts of the Easter Island area (Southeast Pacific Ocean Ridge) associated with a sea urchin

**DOI:** 10.3897/zookeys.1248.159542

**Published:** 2025-08-04

**Authors:** María de los Ángeles Gallardo Salamanca, Cynthia Asorey, Enrique Macpherson

**Affiliations:** 1 Departamento de Biología Marina and Centro de Ecología y Manejo Sustentable de Islas Oceánicas, Universidad Católica del Norte, Larrondo 1281, Coquimbo, Chile Universidad Católica del Norte Coquimbo Chile; 2 Centre d'Estudis Avançats de Blanes (CEAB-CSIC), Carr. Ac. Cala S. Francesc 14, 17300 Blanes, Spain Centre d'Estudis Avançats de Blanes Blanes Spain

**Keywords:** Barcoding, Crustacea, Galatheoidea, symbiosis, taxonomy

## Abstract

*Galatheatukitukimea***sp. nov.** is described from the seamounts near Rapa Nui (Easter Island) and represents the first record of the genus for this region of the Pacific Ocean and for Chilean territory. The new species belongs to the group of species having the carapace with median protogastric and cardiac spines. *G.tukitukimea* has always been observed associated with the sea urchin *Stereocidarisnascaensis*. This potential mimicry-based association is uncommon in squat lobsters, which warrants further study.

## ﻿Introduction

The family Galatheidae contains many species, primarily included in the genus *Galathea* Fabricius, 1793 (e.g. Baba et al. 2008; Macpherson and Baba 2011). The genus is one of the most species-rich of anomuran decapods, containing more than 170 species, most of which are distributed in the Indo-West Pacific Ocean (e.g. Baba et al. 2008; Schnabel et al. 2011; Macpherson and Robainas-Barcia 2015). A significant number of these species have been described in recent years (e.g. Macpherson and Robainas-Barcia 2015; Tiwari et al. 2022, 2024; Rodríguez-Flores et al. 2024; Macpherson et al. 2025) thanks to the increase in sampling efforts (e.g. Richer de Forges et al. 2013; Rodríguez-Flores et al. 2024; Gaymer et al. 2025). These results emphasize that despite the intensive taxonomic efforts, our knowledge gap is still evident in squat lobsters (De Grave et al. 2023).

These species of squat lobsters are found mainly in the Western and Central Pacific but are absent in the Eastern Pacific. The citation of *Galatheapaucilineata* Benedict, 1902, from the Galapagos Islands probably is a species of *Leiogalathea* (Rodríguez-Flores pers. comm.). The easternmost occurrences of *Galathea* in the Pacific are *G.senta* Macpherson & Robainas-Barcia, 2015 from the Pitcairn Islands (Oeno Island, 23°55'S, 130°44'W) and another approximately 15 species from Gambier or the Austral Islands in French Polynesia (Macpherson and Robainas-Barcia 2015).

The seamounts of the Southeastern Pacific have been the focus of increasing sampling effort over the past decades (Fernández et al. 2014; Gaymer et al. 2025), which has facilitated the discovery of numerous new species, including squat lobsters and other groups of benthic invertebrates (Sellanes et al. 2019; Díaz-Díaz et al. 2020; Gallardo et al. 2021; Cañete et al. 2023; Tenorio et al. 2023; de Souza et al. 2024).

During 2024, aboard the RV Falkor (Too), a more thorough sampling of the Salas y Gómez Ridge was carried out in both international waters and within the Chilean Exclusive Economic Zone (EEZ) (FKt240224 Expedition). The technological capabilities of the ROV SuBastian, from the Schmidt Ocean Institute, allowed the discovery of new records and species, as well as the acquisition of HD images that will shed light on ecological associations and habitat use of benthic species. During the expedition, 24 dives were conducted between 200 and 1200 m depth, 11 of them within the Chilean EEZ, including dives around the islands of Rapa Nui and Motu Motiro Hiva, and six seamounts within the Rapa Nui Multiple Use Marine Coastal Protected Area (AMCP-MU Rapa Nui) and the Motu Motiro Hiva Marine Park (MMH Park).

In the present paper, we describe and illustrate, using morphological and molecular data (COI), a new species of *Galathea* collected around AMCP-MU Rapa Nui and in MMH Park and associated with the sea urchin *Stereocidarisnascaensis*Allison et al., 1967. This finding represents the first record of *Galathea* in the Southeastern Pacific and the first documented association between a sea urchin and a species of this genus. To facilitate taxonomic comparisons among morphologically closely related species, we also provide the phylogenetic relationships of some species within the genus *Galathea*.

## ﻿Materials and methods

### ﻿Sampling and measurements

The material examined is in the Museo Nacional de Historia Natural (MNHNC, Santiago, Chile) and Sala de Colecciones Biologicas UCN (SCBUCN, Coquimbo, Chile). The terminology and measurements follow Baba et al. (2011) and Macpherson and Robainas-Barcia (2015). The size of the specimens is indicated by the postorbital carapace length (CL), measured along the midline from the base of the rostrum to the posterior margin of the carapace. The length of the rostrum is measured from the tip to between the lateral basal incisions, the breadth is between left and right lateral basal incisions. Measurements of appendages were taken in dorsal (pereopod 1), lateral (antennule, pereopods 2–4) and ventral (antenna) midlines. Abbreviations used are: Mxp3, maxilliped 3; P1–P4, pereopods 1–4; M = male; F = female; ovig. = ovigerous.

### ﻿Molecular analysis

The DNA extraction, amplification of the cytochrome c oxidase subunit (COI) of the two specimens, and subsequent sequencing were done following the workflow optimized in previous studies on squat lobster’s systematics (e.g. Rodríguez-Flores et al. 2021). DNA was extracted with the DNeasy Blood and Tissue kit (Qiagen), following the manufacturer’s protocol after an overnight digestion. The DNA integrity was assessed on a 2% agarose gel, and its purity was verified by the A260/280 ratio. We used the primers LCO1490 5′ GGTCAACAAATCATAAAGATATTGG-3′ HCO2198 5′-TAAACTTCAGGGTGACCAAAAAATCA-3′ (Folmer et al. 1994; Zuccon et al. 2012), for the partial amplification of the mitochondrial *COI*. PCRs (25 µL total volume) were performed using 2.5 µL of 10 × standard Taq reaction buffer (New England Biolabs), 2 µL of dNTP Mix (2.5 mM), 0.5 µL of each primer (10 mM), 0.125 µL of Taq DNA polymerase (New England Biolabs), 1 µL of BSA (0.06 mg/mL), 1 µL of MgCl_2_ (25 mM), 1 µL of template DNA, and nuclease-free water to complete the final volume. The PCR program consisted of an initial denaturation at 94 °C for 1 min, followed by 5 cycles of 94 °C for 40 s, annealing at 45 °C for 40 s, and extension at 68 °C for 1 min. This was followed by 35 cycles of 94 °C for 40 s, annealing at 57 °C for 40 s, and extension at 68 °C for 1 min, with a final extension step at 68 °C for 5 min. The resulting amplicons were visualized in agarose 1% gels, and the PCR products were sent to Macrogen, Inc. (Seoul, South Korea) for DNA Sanger sequencing.

Forward and reverse raw sequences were aligned, manually edited, and trimmed using Geneious Prime v. 2025.1.2 (https://www.geneious.com). *Galatheatukitukimea* sequences were aligned with other *Galathea* sequences available in GenBank from morphologically closely related species (*G.barbellata*, *G.bicornis*, *G.profunda*, *G.robusta*, and *G.sentosa*). *Galatheatukitukimea* consensus sequences were uploaded to GenBank with accession numbers PV448654–PV448655 (Table 1). Genetic distances for comparisons were estimated using uncorrected ‘p’ divergences in PAUP* v. 4.0 (build 169) (Swofford 2002). COI alignment was performed using MAFFT v. 7.490 (Katoh and Standley 2013) in Geneious Prime with default settings. The COI alignment was used to obtain a phylogenetic reconstruction with the PhyML v. 3.3.2 plugin with the following settings: substitution model = GTR, bootstrap = 1000, the proportion of invariable sites = estimated, gamma distribution parameter = estimate, optimize = Topology/length/rate (Guindon et al. 2010).

**Table 1.** Species included in the present study, with COI GenBank accession number, locality, biogeographic region, and collection depth.

Species	GenBank accession no. (COI)	Locality	Biogeographic region	Depth (m)	Reference
*Galatheabarbellata*	KP203589.2	Vanuatu and New Caledonia	Southwest Pacific Ocean	182–270	Macpherson and Robainas-Barcia 2015
*Galatheabicornis*	PQ423191	New Caledonia	Southwest Pacific Ocean	81	Macpherson et al. 2025
*Galatheaprofunda*	KP203662.2	Vanuatu and New Caledonia	Southwest Pacific Ocean	270–702	Macpherson 2012
*Galathearobusta*	KP203663.2	Madagascar, La Réunion, Mauritius	Western Indian Ocean	105–238	Macpherson and Robainas-Barcia 2015
*Galatheasentosa*	KP203664.2	Wallis and Futuna Islands	Southwest Pacific Ocean	245–440	Macpherson and Robainas-Barcia 2015
*Galatheatukitukimea* sp. nov.	PV448654	Motu Motiro Hiva	Southeastern Pacific Ocean	407	This study
*Galatheatukitukimea* sp. nov.	PV448655	Pukao seamounts	Southeastern Pacific Ocean	348	This study
*Grimotheamonodon* (outgroup)	AY351062.1	Chile Continental	Southeastern Pacific Ocean	94–523	Machordom and Macpherson 2004

### ﻿Systematic account

**Superfamily Galatheoidea Samouelle, 1819**

**Family Galatheidae Samouelle, 1819**

**Genus *Galathea* Fabricius, 1793**

#### ﻿ Galathea tukitukimea sp. nov.

Taxon classificationAnimaliaDecapodaGalatheidae

F2ED9100-5771-5124-B666-B92C1A642810

https://zoobank.org/906F5BDA-B2FD-43F4-94C3-2E31E7219838

Figs 1 , 2

##### Material examined.

Salas and Gómez Ridge. ***Holotype***: • Motu Motiro Hiva (Salas and Gómez Island). Cruise FKt240224, Stn 671-BB1A-005, 26°28'35"S, 105°13'58"W, 407 m, 20 March 2024: M 3.3 mm (MNHNC DEC-15582). ***Paratype***: • Pukao seamount. Cruise FKt240224, Stn 667-BB1D-002, 26°54'52"S, 110°14'26"W, 348 m, 16 March 2024: ovig. F 4.4 mm (SCBUCN6722).

##### Etymology.

The specific epithet *tukitukimea* derives from the Rapa Nui words *tuki tuki mea*, meaning “red dots”, in reference to the vivid reddish spots on the carapace and pereiopods. The name was proposed by Serafina Moulton Tepano, a Rapa Nui artist who accompanied the FKt240224 expedition. It is treated as a noun in apposition.

##### Description.

***Carapace***: longer than broad; anterior and posterior cervical grooves distinct; dorsal surface with scale-like and interrupted ridges in all regions; mid-transverse ridge laterally interrupted and not scale-like, preceded by distinct cervical groove; transverse groove before cardiac spines; ridges not densely setose, with short simple setae (sometimes with a few short setules) and with some long and median thick, plumose setae on protogastric and cardiac ridges. Epigastric region with 2 spines and 1 or 2 minute acute granules; 2 median protogastric spines; 1 lateral protogastric and 1 parahepatic spine on each side; 2 median metagastric spines. Cardiac region with 2 median spines. Branchial regions each with 1 anterior branchial 2 posterior spines. Lateral margins slightly convex, with 8 spines: 3 spines in front of and 5 spines behind anterior cervical groove; first anterolateral, well developed, posterior to level of lateral limit of orbit; second and third spines small, situated at midlength between anterolateral spine and anterior cervical groove; 2 spines on anterior branchial region, second very small, and 3 spines on posterior branchial margin, third very small; 1 additional spine below lateral margin, between first and second anterolateral spines. Outer orbital angle acute; infra-orbital margin with 1 or 2 spines. Rostrum 2.7 × as long as broad, length 0.8 that of, breadth 0.3 that of carapace; distance between distalmost lateral incisions 0.4 distance between proximalmost lateral incisions; dorsal surface nearly horizontal in lateral view, with minute setiferous ridges; lateral margin with 4 sharp spines.

Pterygostomian flap rugose with sparse setae, anteriorly rounded; some granules on upper margin near linea anomurica.

***Pleon***: somites 2 and 3 each with 2 uninterrupted transverse ridges on tergite; somite 4 with 1 uninterrupted and 1 medially interrupted transverse ridge; somites 5 and 6 each with scale-like ridges, each tergite with some long and thick plumose setae; posteriormedian lobe of somite 6 indistinct. Telson incompletely subdivided. Two pairs of male gonopods.

***Thoracic sternum***: 1.2 × as long as wide. Sternite III with median shallow notch. Sternite IV with anterior part narrower than sternite III, with some short striae. Sternites IV–VI with a few striae. Sternite III ~1.7 × as wide as long; sternite IV nearly 2.1 × as wide as long, and 3.5 × as wide as sternite III.

***Eye***: ocular peduncles 1.8 × longer than broad, maximum corneal diameter 0.7 of rostrum width.

***Antennule***: article 1 with 3 distal spines, distomesial smaller than distolateral, distodorsal spine larger than others; lateral margin with 2 small spines.

***Antenna***: article 1 with distomesial spine not reaching distal margin of article 2. Article 2 with distomesial shorter than distolateral spine, not reaching end of article 3. Articles 3 and 4 unarmed.

***Mxp3***: ischium with spine on extensor and flexor distal margins. Merus subequal in length to ischium, with strong median and small distal spine on flexor margin; extensor margin with small distal spine. Carpus smooth along extensor margin.

***P1***: 3.3 × carapace length, relatively slender, somewhat depressed on palm, more so on fingers. Merus 1.4 × as long as carapace, 3 × as long as carpus, with spines arranged roughly in rows, distal spines prominent. Carpus 3.4 × as long as palm, 1.6 × longer than broad; dorsal surface with small spines arranged roughly in longitudinal rows; mesial margin with 2 or 3 strong spines. Palm 4.3 × longer than broad, lateral and mesial margins subparallel; spines arranged roughly in rows; dorsolateral row continuing onto lateral margin of fixed finger. Fingers 0.4 × as long as palm; movable finger ending in a slightly curved spine; flexor margin with 6 or 7 serrated distal teeth, and a strong subtriangular tooth proximally, finely setose.

***P2–4***: moderately slender. P2 1.8 × carapace length. Meri successively shorter posteriorly (P3 merus 0.8 length of P3 merus, P4 merus 0.7 length of P3 merus); P2 merus 0.7 of carapace length, 4.5 × as long as broad, 1.5 × longer than P2 propodus. Extensor margin with row of 5 or 6 proximally diminishing spines on P2 and P3, 2 or 3 distal spines on P4; ventral margins distally ending in strong spine followed proximally by 2 or 3 spines and several eminences. Carpi with 5 or 6 spines on extensor margin on P2–4; lateral surface with 3 or 4 spines or acute granules subparallel to extensor margin; flexor distal margin acute. Propodi 3.5–4.0 × as long as broad; extensor margins with 3 or 4 proximal spines; flexor margin with 6–8 slender movable spines, terminal spines paired. Dactyli distally ending in well-curved, strong spine, length 0.5–0.6 that of propodi; flexor margin with 5 proximally diminishing teeth, terminal tooth prominent.

Epipods present on P1, absent on P2 and P3.

##### Genetic data.

COI. 671-BBIA-005, GenBank code: PV448654. 667-BBID-002, GenBank code: PV448655.

##### Remarks.

The new species belongs to the species group having the carapace with paired median protogastric and cardiac spines. This group now contains eight species: *G.barbellata* Macpherson, 2012 from New Caledonia, *G.bicornis*Macpherson et al., 2025 from New Caledonia, *G.echinata* Macpherson, 2012 from New Caledonia, *G.profunda* Macpherson, 2012 from New Caledonia and Vanuatu, *G.robusta* Baba, 1990 from Madagascar, *G.sentosa* Macpherson & Robainas-Barcia, 2015 from Wallis and Futuna, and the new species from Salas and Gómez Ridge. The new species can be distinguished from these species by the following characters:

- *Galatheabicornis*, *G.echinata*, *G.robusta*, and *G.sentosa* have the antennular article 1 with 2 well-developed distal spines, with the distomesial spine minute or obsolescent, whereas the new species has 3 well-developed distal spines.
- Only 3 species of the group (*G.barbellata*, *G.robusta*, and the new species) have 3 well-developed distal spines on the antennular article 1: *G.barbellata* and *G.profunda* both have the metagastric region unarmed, whereas there are 2 spines in the new species. Furthermore, the branchial regions are unarmed in *G.barbellata* and *G.profunda*, whereas these regions have some spines in the new species. Finally, the epigastric region is armed with numerous spines in *G.barbellata* and unarmed in *G.profunda*, whereas this region has only 2 spines.
- The rostrum is much larger in the new species than in *G.barbellata*.

*Galatheatukitukimea* showed the lowest COI genetic divergence with *G.profunda* (12.0%). Concerning the other morphologically similar species, the divergences ranged from 16.8% (*G.sentosa* and *G.barbellata*), 17.8% (*G.bicornis*) to 19.9% (*G.robusta*) (unfortunately no COI data are available for *G.echinata*).

##### Coloration.

**Figure 1.**
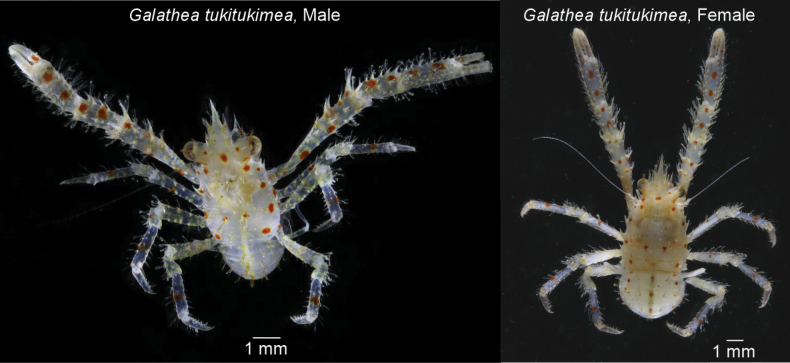
*Galatheatukitukimea* sp. nov., holotype, male (MNHNC DEC-15582) and paratype, female (SCBUCN6722). Color in life, dorsal view.

Dorsal surface of the cephalothorax, chelipeds, and pereiopods white, with yellow-orange blotches bearing red spots. Dorsal regions covered with iridescent setae. Ventral surfaces of pereiopods and chelipeds exhibit the same coloration pattern. Sternite white (Fig. 1).

##### Distribution.

Chilean Exclusive Economic Zone; Rapa Nui Multiple-Use Coastal Marine Protected Area (AMCP-MU Rapa Nui); on the Pukao seamounts; Motu Motiro Hiva Marine Park (MMH Park); around to Motu Motiro Hiva Island (Salas and Gómez Island), at depths of 348–407 m.

**Figure 2.**
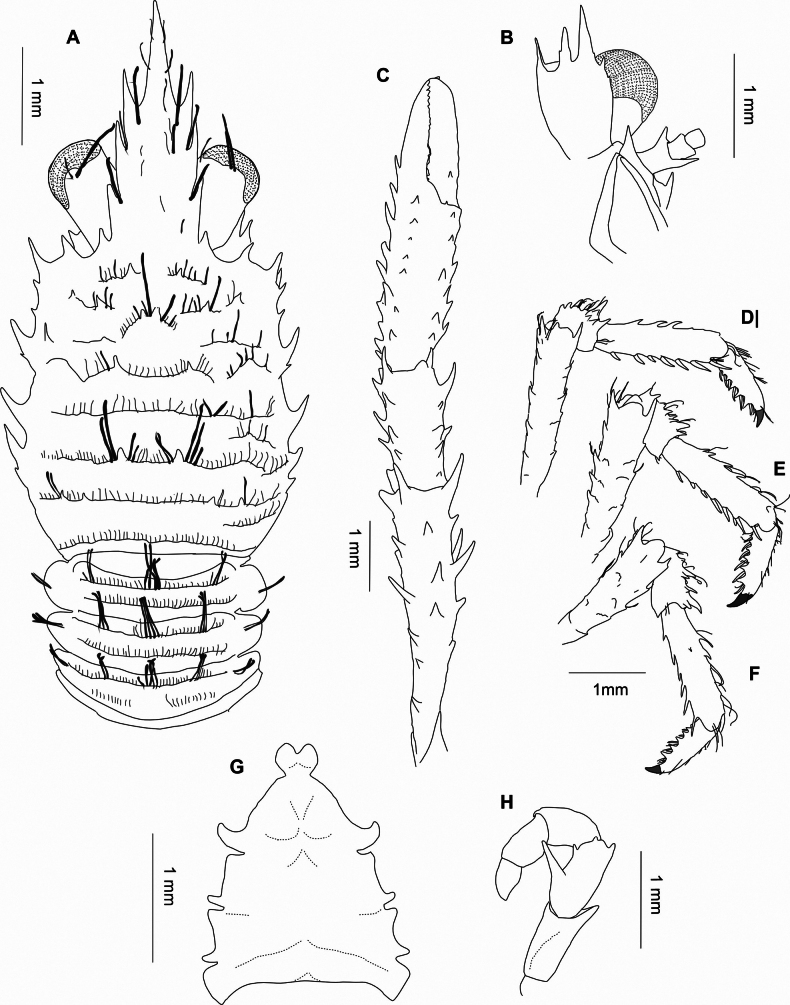
*Galatheatukitukimea* sp. nov., MNHNC DEC-15582, holotype, male. A. Carapace and pleonal tergite 1–4, dorsal view; B. Left antennular and antennal peduncles, ventral view; C. Right P1, the setation of all articles is omitted; palm, merus, and carpus bear long plumose setae on the extensor margin, and the distal region of the manus is finely setose; D–F. Left P2-P4, lateral view; G. Sternal plastron, ventral view; H. Left third maxilliped, ventral view.

##### Habitat and ecology.

**Figure 3.**
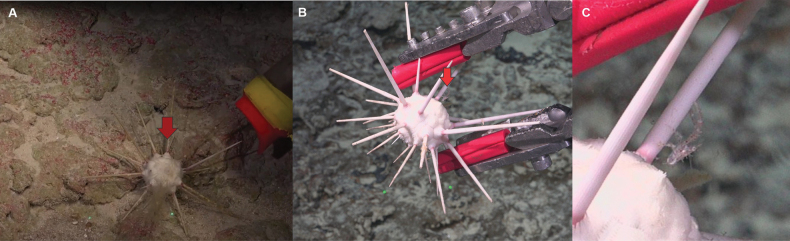
*Galatheatukitukimea* sp. nov. on *Stereocidarisnascaensis* in habitat. A. Pukao seamount, depth 348 m; B. Motu Motiro Hiva Island, depth 407 m; C. *G.tukitukimea* on the spine of *S.nascaensis*. Image credit ROV SuBastian/Schmidt Ocean Institute, FKt240224 expedition.

Specimens of *G.tukitukimea* were living among the perianal spines of the sea urchin *Stereocidarisnascaensis* (Fig. 3). This urchin is white with a reddish tone at the base of its spines. The sea urchins were found on rocky bottoms, and on the Pukao seamounts, the substrate was covered with red crustose algae at a depth of 348 m.

##### Ocean census species number.

OC186.

## ﻿Discussion

The discovery of *Galatheatukitukimea* in the southeastern Pacific represents the first record of the genus from this region of the ocean and from Chilean territory. This finding supports the hypothesis pointed out in other studies (e.g. Gallardo et al. 2021) of a biogeographic connectivity and faunal affinity between the Salas and Gómez Ridge and the subtropical communities of the western and central Pacific. In fact, during the preliminary study of the squat lobsters found in the recent expeditions (e.g. FKT240224) we have observed a clear overlap among the species found in Salas and Gómez and Nazca Ridges and the southwestern and central Pacific fauna (manuscript in preparation).

Sampling carried out in the seamounts of the southeastern Pacific are demonstrating the existence of a little-known fauna (Mecho et al. 2021; Wagner et al. 2021; Gaymer et al. 2025). Likewise, these samples carried out with ROV also allow us to know quite reliably the habitat where many of these species are found (Asorey et al. 2020; Gallardo et al. 2021; Tapia-Guerra et al. 2021; Cañete et al. 2023). The new species found in the seamounts of Salas and Gómez Ridge has always been observed associated with the sea urchin *Stereocidarisnascaensis*. This sea urchin is distributed in the southern Desventuradas Islands, the intersection of the Nazca and Salas and Gómez Ridges, and along Salas and Gómez ridges (Mecho et al. 2021). This expedition extended the distribution range of *S.nascaensis* westward, within the Easter Island ecoregion. This potential mimicry-based association is uncommon in squat lobsters, which warrants further study. Previous records of *S.nascaensis* were obtained using Agassiz trawls (Parin et al. 1997; Mecho et al. 2021), a more aggressive sampling method that may have prevented the simultaneous collection of *Galathea* specimens. In contrast, the new records were made using the manipulator arm of the ROV *SuBastian*, a more precise and less disruptive technique.

Most of the symbiotic relationships between squat lobsters and macro-invertebrates have been observed with soft and black corals (see the review by Baeza 2011). In the species of Galatheidae, the association with echinoderms is common among crinoids and species of the genus *Allogalathea* (Cabezas et al. 2011) and *Galathea* (Baba and Fujita 2008; Baba et al. 2009). An association between squat lobsters and sea urchins has only been observed between *Munidopsisabdominalis* (family Munidopsidae) and echinoids of the genus *Cidaris* (Rice and Miller 1991).

**Figure 4.**
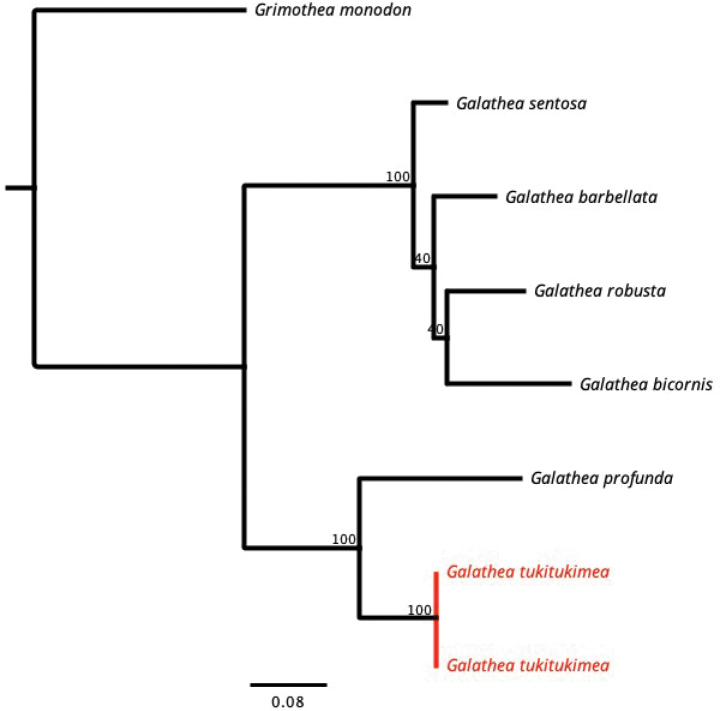
Phylogenetic tree obtained after the maximum-likelihood analysis based in COI sequences.

Morphologically, *Galatheatukitukimea* is related to other species of the genus distributed from Madagascar to New Caledonia and Wallis and Futuna, e.g. *G.barbellata*, *G.bicornis*, *G.echinata*, *G.profunda*, *G.robusta*, and *G.sentosa* (e.g. Baba 1990; Macpherson 2012). These species belong to the same molecular clade (Fig. 4), but only a single gene (COI) is available, so their phylogenetic relationships cannot be known reliably. Most of the morphologically related species inhabit deep waters between 100 and 700 m depth, although most species of the genus *Galathea* inhabit shallow waters (<100 m) (Baba et al. 2008; Schnabel et al. 2011), which suggests some common ecological characteristics. The genetic divergence in the COI gene between *G.tukitukimea* and six of these seven species ranges from 12% to 20.5%, which falls within the range previously reported for the genus *Galathea* (Macpherson and Robainas-Barcia 2015). These genetic distances support the distinctiveness of *G.tukitukimea* within this group, though further molecular markers and ecological data are necessary to resolve their phylogenetic relationships more robustly. Obviously, more data, both molecular and ecological, are needed to establish a more complete phylogeny of this group of species.

## Supplementary Material

XML Treatment for Galathea tukitukimea

## ﻿Additional information

### Use of AI

No use of AI was reported.

### Author ORCIDs

Cynthia Asorey https://orcid.org/0000-0002-4777-421X

### Data availability

All of the data that support the findings of this study are available in the main text.
